# Supplementary material for: Epitaxially grown silicon-based single-atom catalyst for visible-light-driven syngas production
Source: Nat Commun. 2023 Mar 28;14:1719. doi: 10.1038/s41467-023-37401-3 (PMC10050177; doi:10.1038/s41467-023-37401-3)
Supplement: Supplementary file 1 — Supplementary Information [file 41467_2023_37401_MOESM1_ESM.pdf]

Supplementary Materials for

Epitaxially grown silicon-based single-atom catalyst for visible-light-driven syngas production

Huai Chen,[1†] Yangyang Xiong,[1†] Jun Li,[2,3] Jehad Abed,[2] Da Wang,[4,5] Adrián Pedrazo-Tardajos,[4,5] Yueping Cao,[1] Yiting Zhang,[1] Ying Wang,[6] Mohsen Shakouri,[7] Qunfeng Xiao,[7] Yongfeng Hu,[7] Sara Bals,[4,5] Edward H. Sargent,[2] Cheng-Yong Su,[1*] Zhenyu Yang[1*]

[1] MOE Laboratory of Bioinorganic and Synthetic Chemistry, Lehn Institute of Functional Materials, School of Chemistry, Sun Yat-sen University, Guangzhou, 510275, China

[2] Department of Electrical and Computer Engineering, University of Toronto, 35 St. George Street, Toronto, Ontario M5S 1A4, Canada

[3] Frontiers Science Center for Transformative Molecules, Shanghai Jiao Tong University, Shanghai 200240, China

[4] Electron Microscopy for Materials Science (EMAT), University of Antwerp, Groenenborgerlaan 171, 2020 Antwerp, Belgium

[5] NANOlab Center of Excellence, University of Antwerp, 2020 Antwerp, Belgium

[6] Department of Chemistry, Chinese University of Hong Kong, Shatin, New Territories, Hong Kong SAR

[7] Canadian Light Source, Inc. (CLSI), Saskatoon, Saskatchewan, Canada

† These authors contributed equally to this work.

E-mail: cesscy@mail.sysu.edu.cn (C.S.); yangzhy63@mail.sysu.edu.cn (Z.Y.)

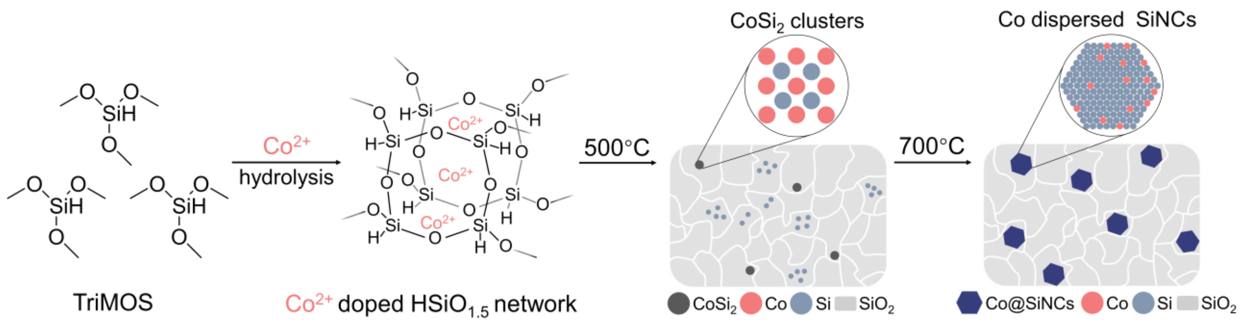

Supplementary Fig. 1 | Schematic of the synthetic steps of Co@Si SACs embedded in amorphous SiO_2.

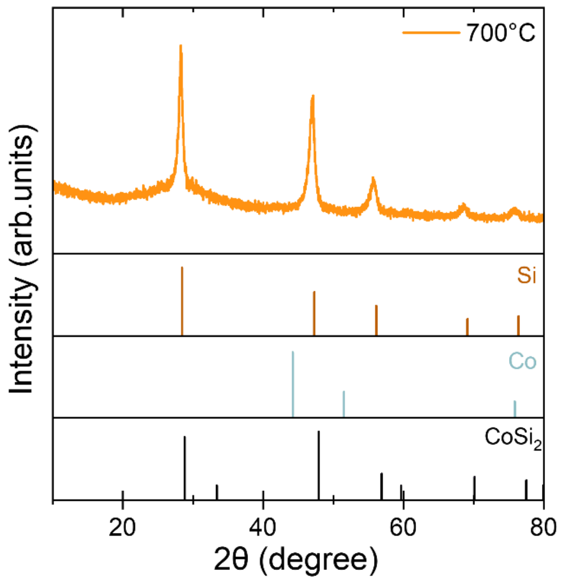

Supplementary Fig. 2 | Powdery XRD pattern of free-standing Co@Si NCs obtained from HF etching of the solid of Co@Si SACs with 0.46 wt%Co in SiO_2 synthesized at 700 ^oC. The particle size is 12.3 ± 2.55 nm, estimated using Scherrer analysis. JCDPS Reference: Si: #27-1402; Co: #15-0806; $CoSi_2$: #38-1449.

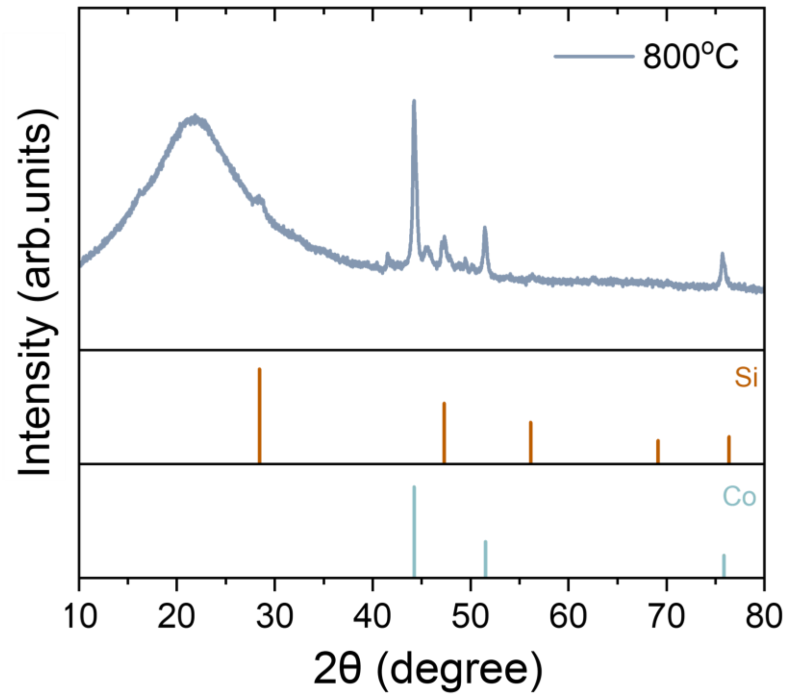

Supplementary Fig. 3 | XRD result of the Co@Si sample annealed at 800°C for 12 h. JCDPS Reference: Si: #27-1402; Co: #15-0806.

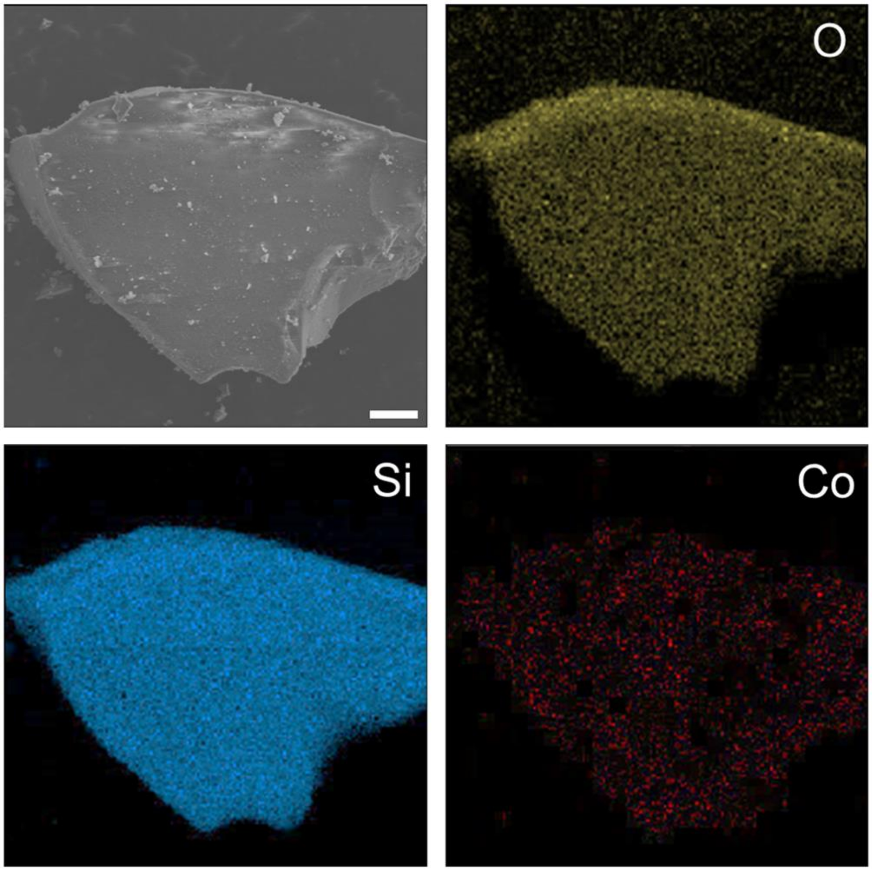

Supplementary Fig. 4 | SEM image and the corresponding energy dispersive X-ray spectroscopy (EDX) mapping results of the powders of Co@Si embedded in SiO_2 (scale bar = 10 μm, Co concentration: 1.4 wt%).

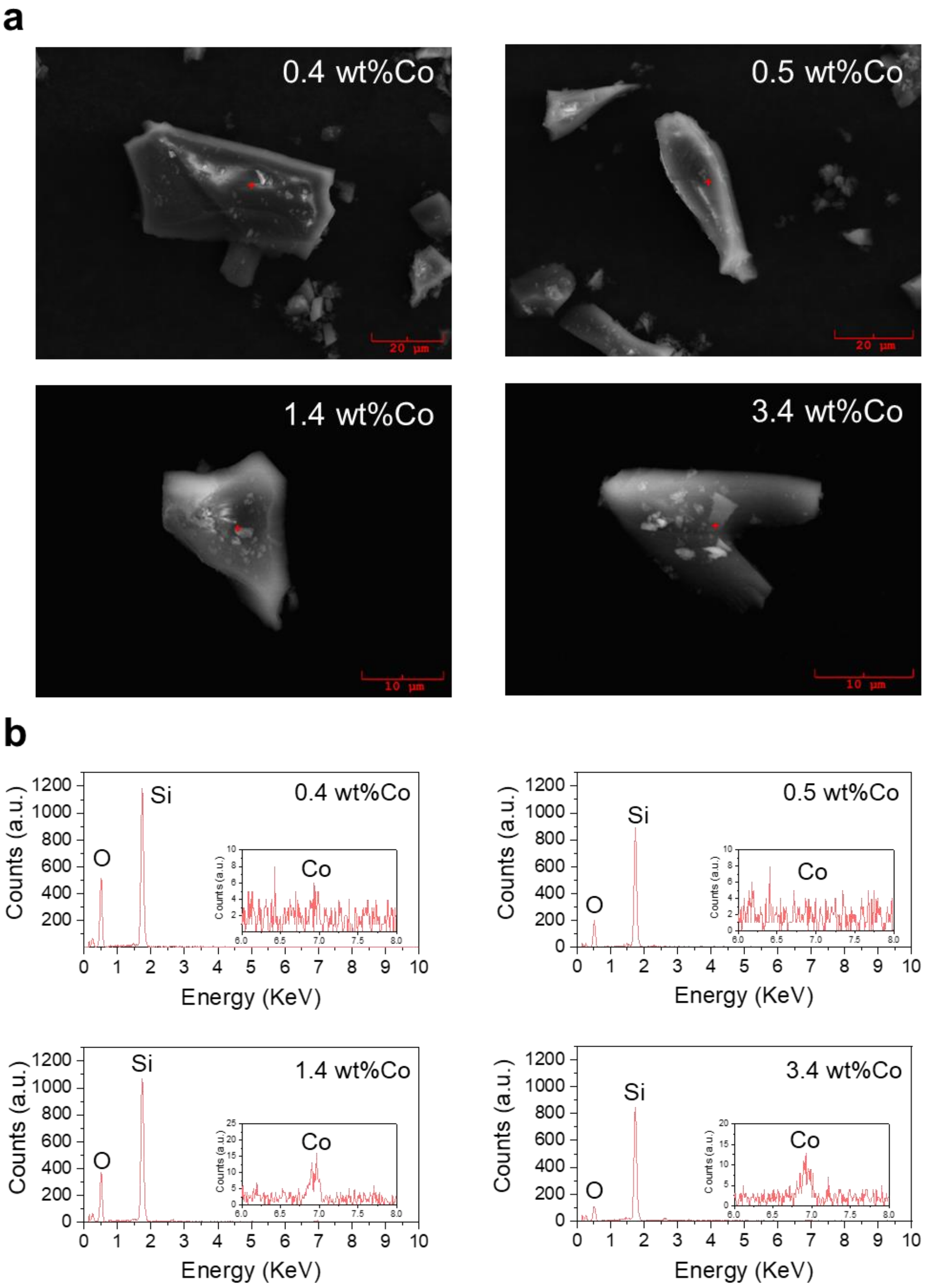

Supplementary Fig. 5 | SEM and EDX results of Co@Si SACs. a, SEM images and **b,** the corresponding EDX spectra of the powders of Co@Si embedded in SiO_2 with various Co concentrations highlighted on the SEM images. Inset: zoom-in EDX results showing the presence of Co signals.

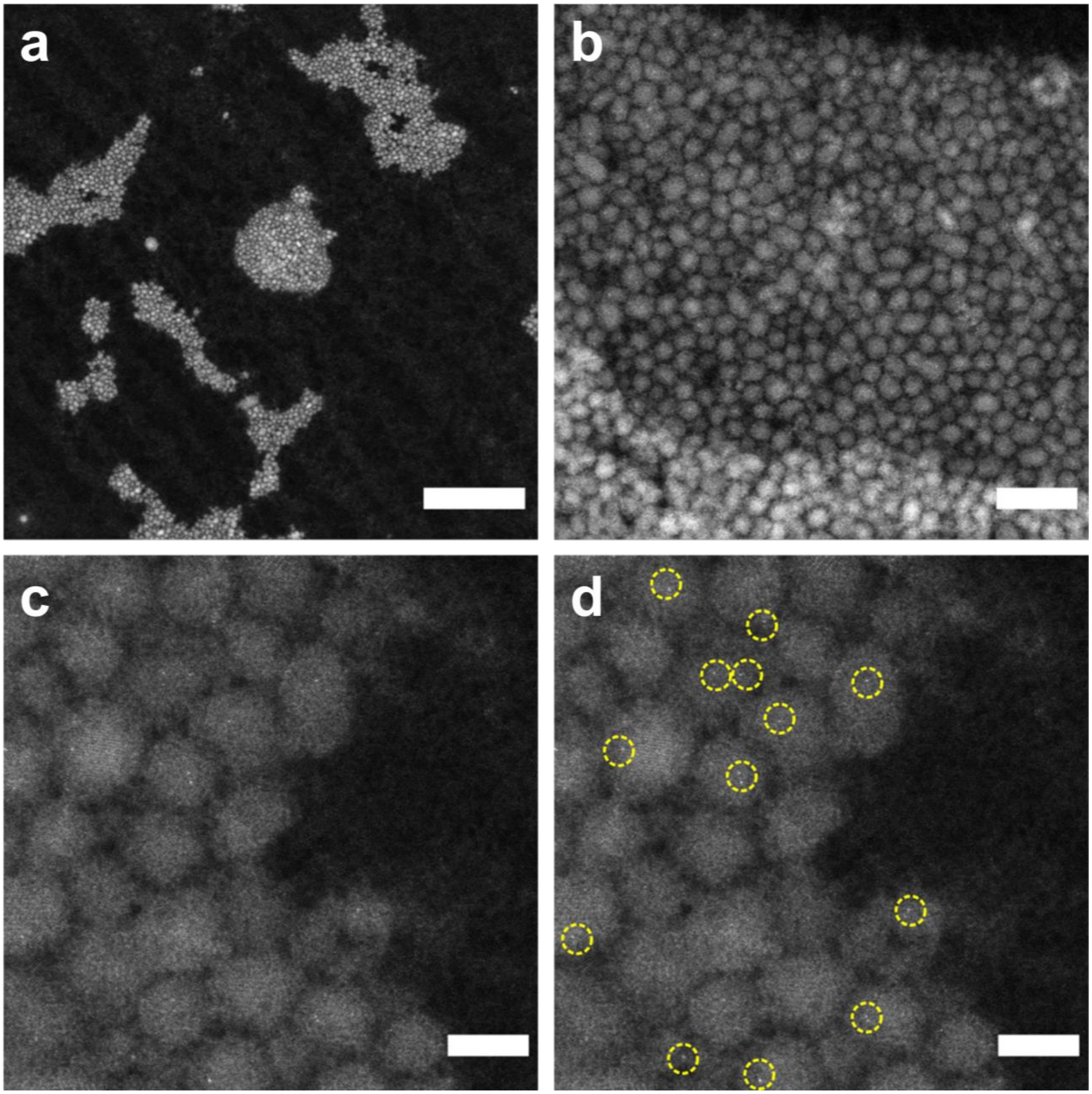

Supplementary Fig. 6 | Additional HAADF-STEM images of ligand-functionalized Co@Si SACs. a-c, HAADF-STEM images of ligand-functionalized Co@Si NCs at different magnifications. **d,** same areas as shown in panel **c** with yellow dashed circles, denoting the locations where the Co atoms are likely present (scale bars: (a) 100 nm, (b) 20 nm, (c-d) 5 nm).

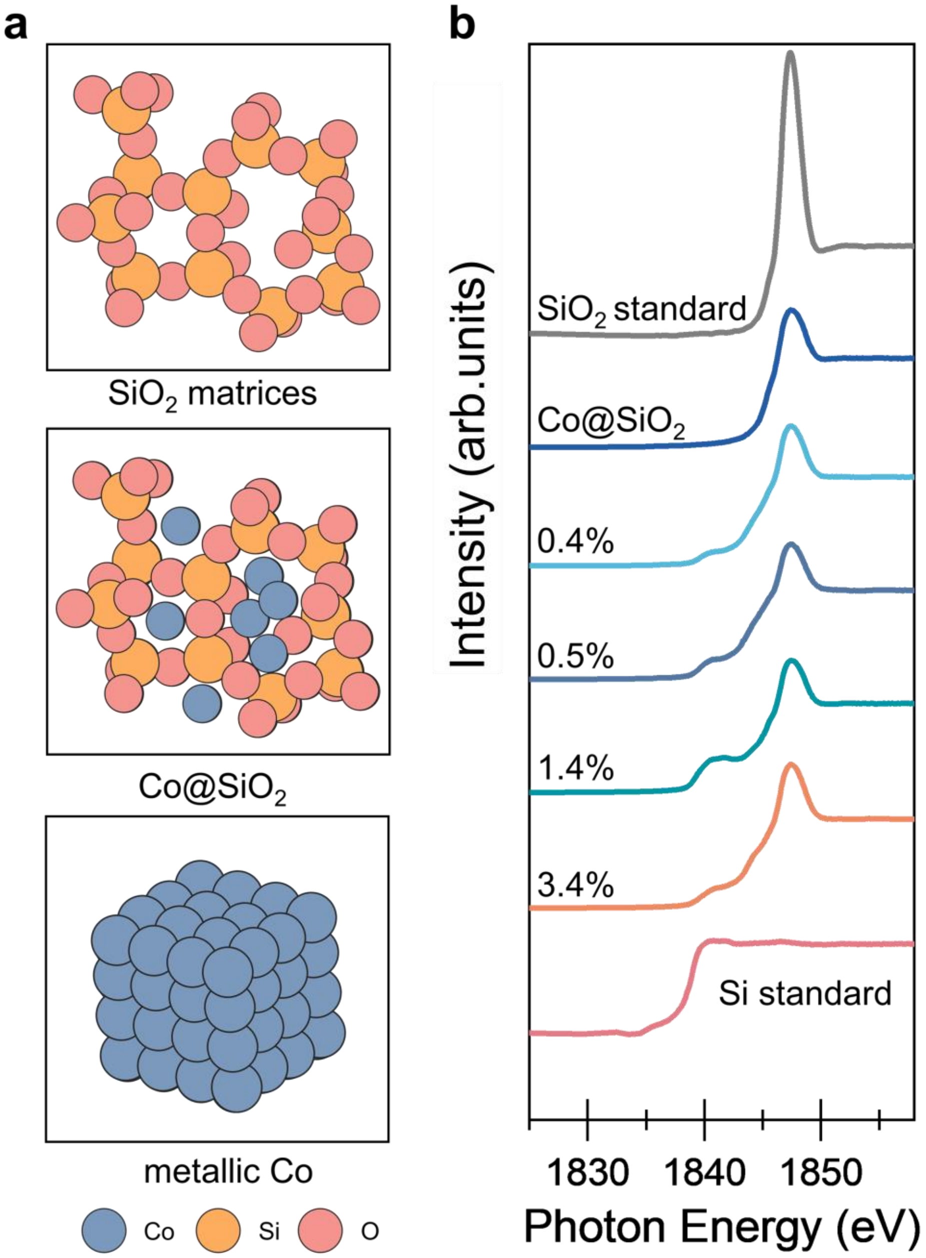

Supplementary Fig. 7 | XANES results of Co@Si SACs and Co@SiO_2 control sample. a, schematics of three types of control samples: SiO_2 matrices (i.e., amorphous SiO_2-only sample), Co@SiO_2 (i.e., Co atoms dispersed in amorphous SiO_2), and metallic Co. **b,** X-ray absorption near-edge structure (XANES) spectra at the Si K-edge of Co@Si SACs with different Co concentrations, standard materials (i.e., Si and amorphous SiO_2), and the control sample Co@SiO_2.

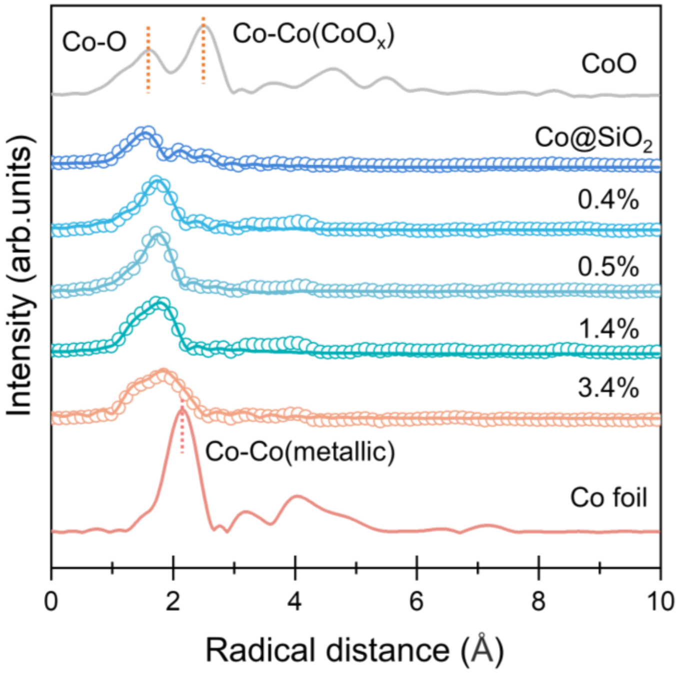

Supplementary Fig. 8 | The original Co K-edge EXAFS spectra (circles) and the corresponding fitting results of various Co@Si and control samples as shown in Fig. 3b.

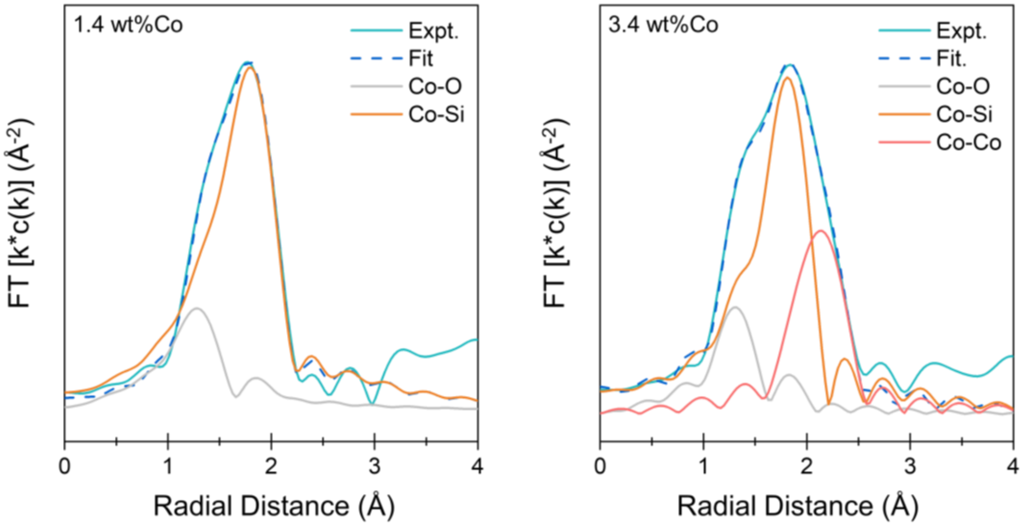

Supplementary Fig. 9 | The Co K-edge EXAFS spectra and the corresponding fitted curves together with scatter paths from EXAFS fitting of Co@Si samples at Co loadings of 1.4% and 3.4%. The detailed fitted results are listed in Supplementary Table 3.

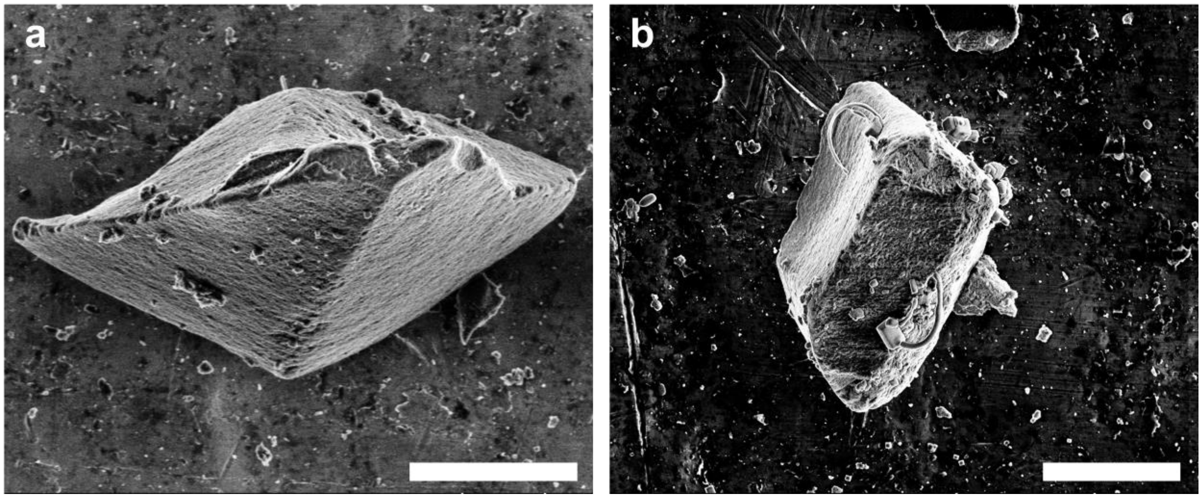

Supplementary Fig. 10 | SEM images of Co@Si SACs in SiO_2 before and after the photocatalytic reaction. a, Co@Si SAC with 0.5 wt%Co in SiO_2 before the irradiation and **b,** after the white light irradiation (λ_{ex} > 400 nm; irradiation time: 3 h; scale bar: 5 μm).

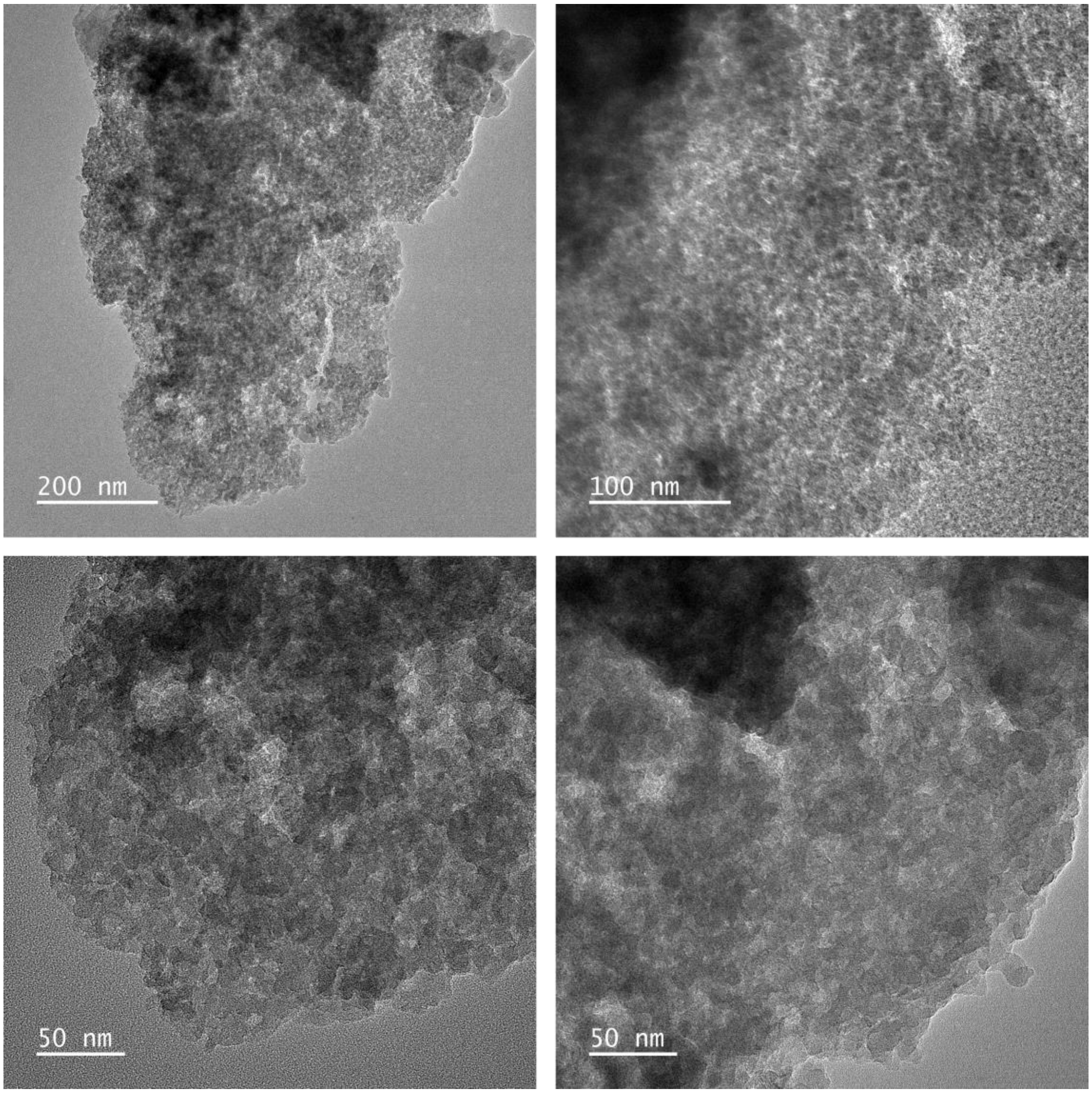

Supplementary Fig. 11 | Bright-field TEM images of Co@Si SAC sample with 3.4wt%Co.

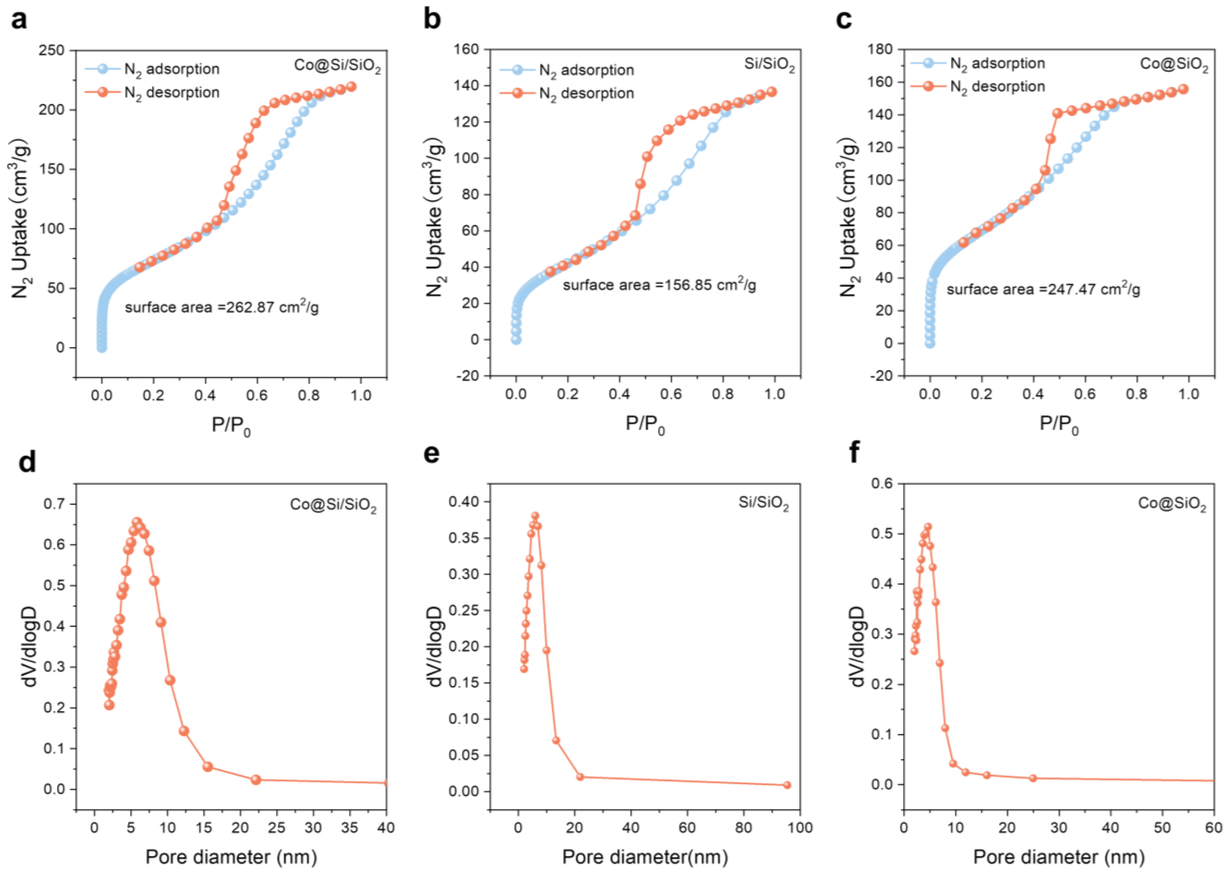

Supplementary Fig. 12 | BET isotherm results of Co@Si SAC and control samples. a-c, N_2 adsorption/desorption isotherms of Co@Si SAC (denoted as "Co@Si/SiO_2 for clarity) with 3.4 wt%Co, and control samples (Co-free Si/SiO_2 and Co@SiO_2) measured at 77 K. **d-f,** the corresponding pore size distributions of three samples based on the Barrett-Joyner-Halenda (BJH) methods of N_2 absorption curves in Fig. a-c (Note: V = pore volume, D = pore diameter).

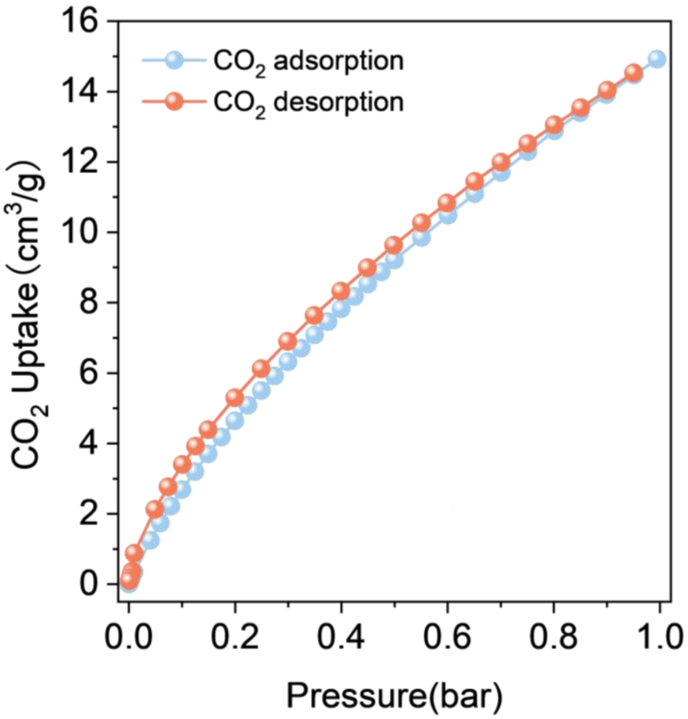

Supplementary Fig. 13 | CO_2 isotherm adsorption and desorption curves of Co@Si SAC with 3.4 wt%Co in SiO_2 measured at 273 K.

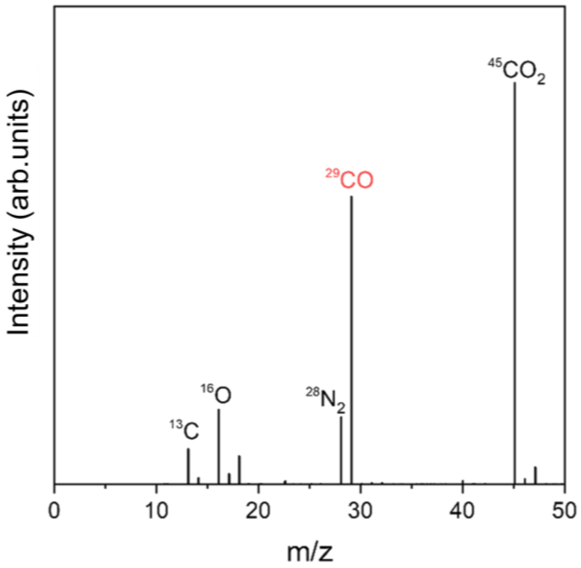

Supplementary Fig. 14 | Gas chromatography-mass spectrometry (GC-MS) analysis of ^{13}CO generated from the photocatalytic reduction of $^{13}CO_2$ by Co@Si SAC with 0.5 wt%Co. The signals of ^{13}C and ^{16}O originate from the fractions of ^{29}CO and O_2. The component of O_2 and N_2 may result from the gas leakage by injection.

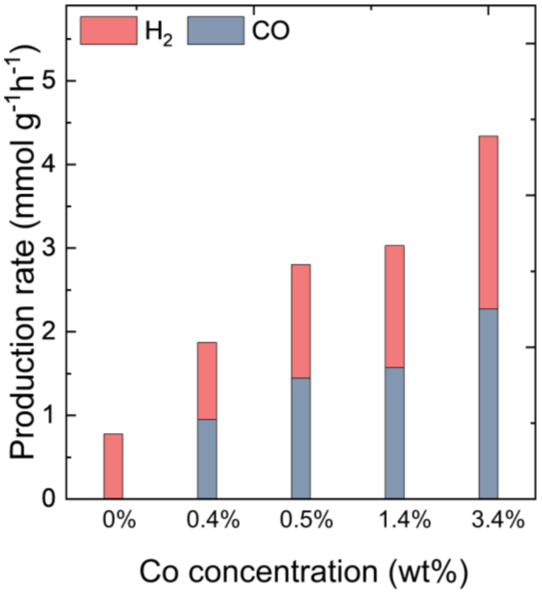

Supplementary Fig. 15 | Photocatalytic performance of the SACs with different Co concentrations. 2 mg of Co@Si SACs in SiO_2 and the control sample Co-free Si/SiO_2 were applied for each reaction proceeded for 6 h.

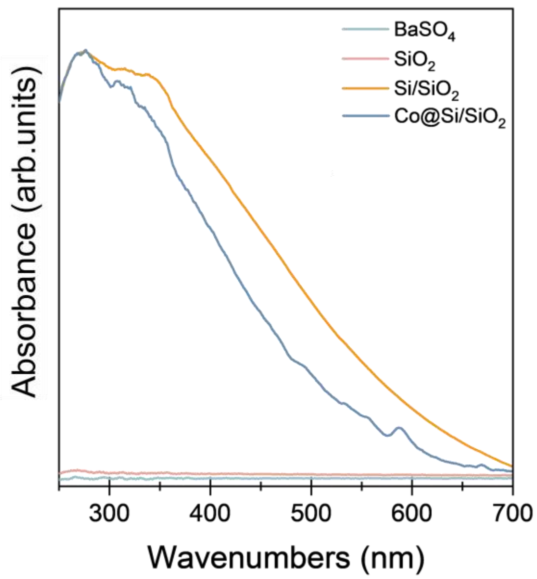

Supplementary Fig. 16 | Absorption spectra of Co@Si SAC with 0.5 wt%Co powders and control samples (i.e., $BaSO_4$ substrate, SiO_2, and Co-free Si/SiO_2 powders). The Co@Si SAC sample is denoted as "Co@Si/SiO_2" to highlight the presence of the SiO_2 matrix.

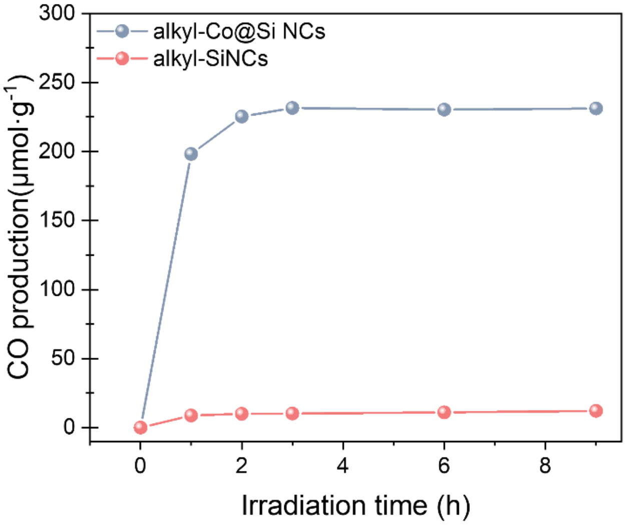

Supplementary Fig. 17 | Comparison of the CO production performance between HF-etched Co@Si (i.e., after removing the SiO_2 matrix) and Co-free Si NCs.

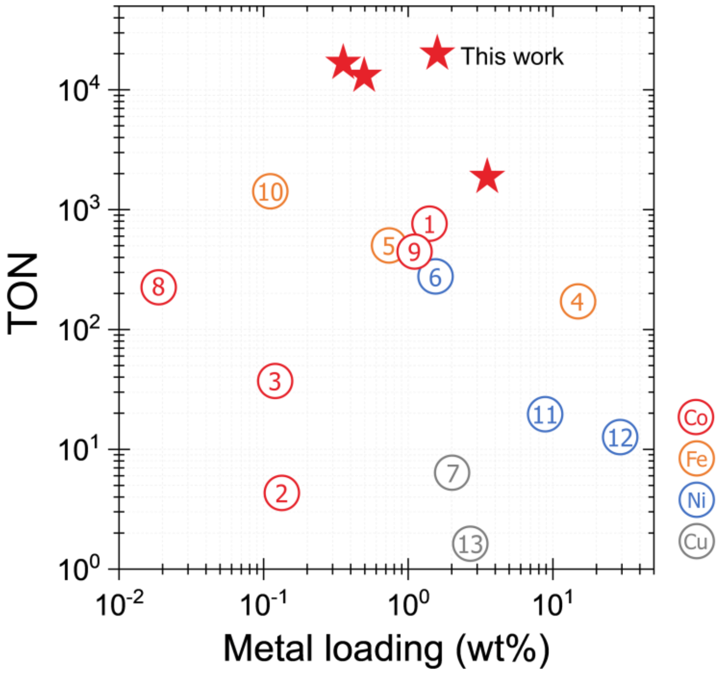

Supplementary Fig. 18 | The comparison of the TON values and metal loading results of the reported photocatalysts. The reference list is available in Supplementary Table 4.

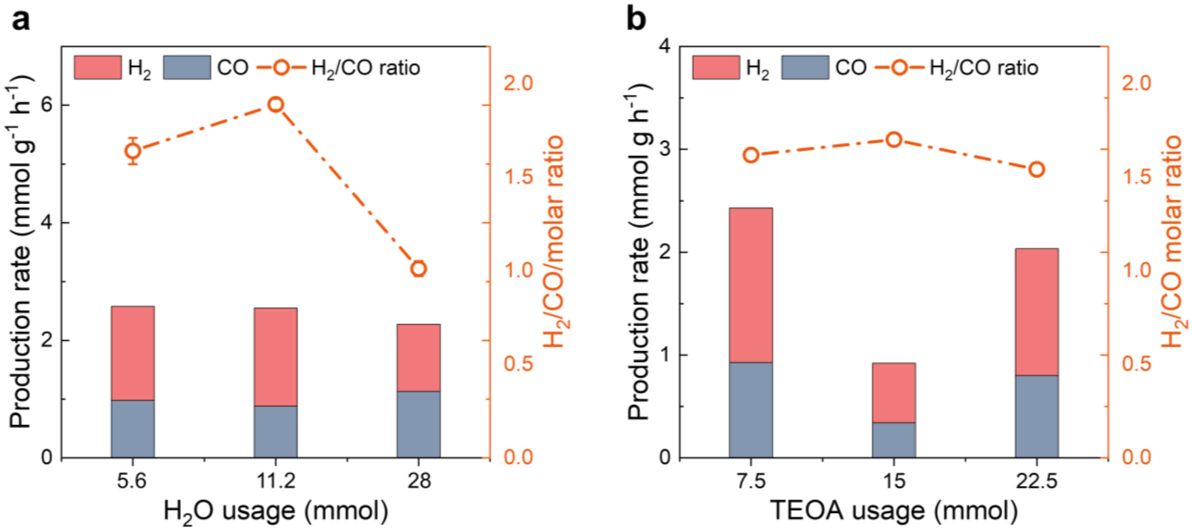

Supplementary Fig. 19 | Photocatalytic performance of the Co@Si SAC with 0.5 wt%Co with the presence of different amounts of (a) water and (b) TEOA. 7.5 mmol TEOA and 5.6 mmol H_2O was used consistently in (a) and (b), respectively. 2 mg of Co@Si SAC sample was used for each reaction (irradiation time: 3 h). The error is due to the deviation of multiple measurements. The detailed discussion about the influences of H_2O and TEOA on the H_2/CO ratio in the syngas

product is available in the Supplementary Note 2.

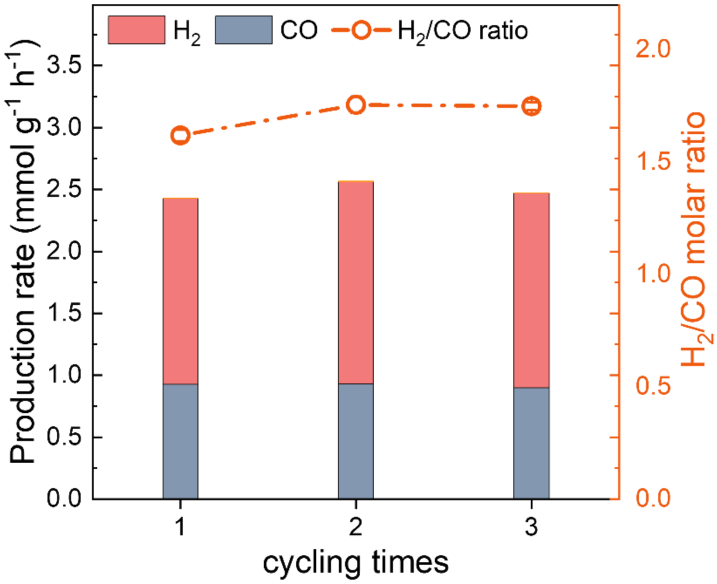

Supplementary Fig. 20 | Photocatalytic cyclability of Co@Si SACs with 0.5 wt%Co. 2 mg of Co@Si SAC in SiO_2 were used for each reaction (irradiation time: 3 h).

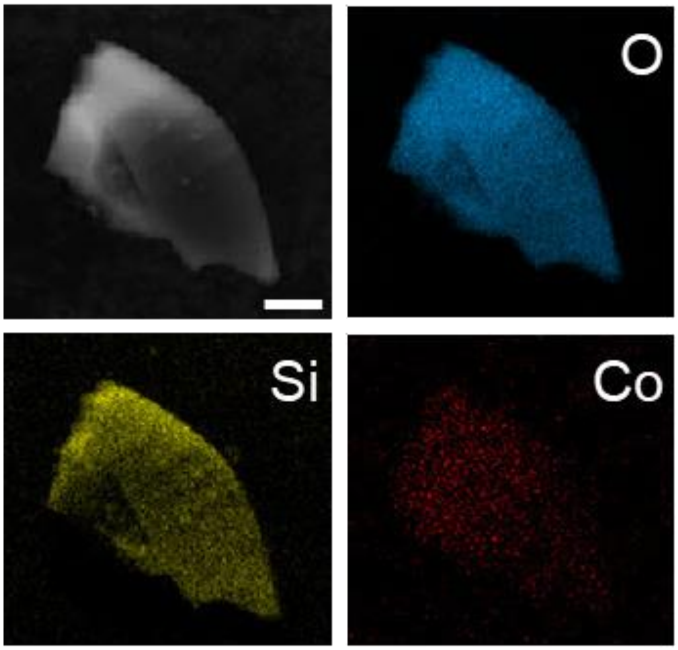

Supplementary Fig. 21 | SEM image of Co@Si SAC 1.4 wt%Co in SiO_2 after 3 h irradiation and the corresponding elemental distribution of Si, O, and Co verified by EDX mapping

(scale bar: 2 μm).

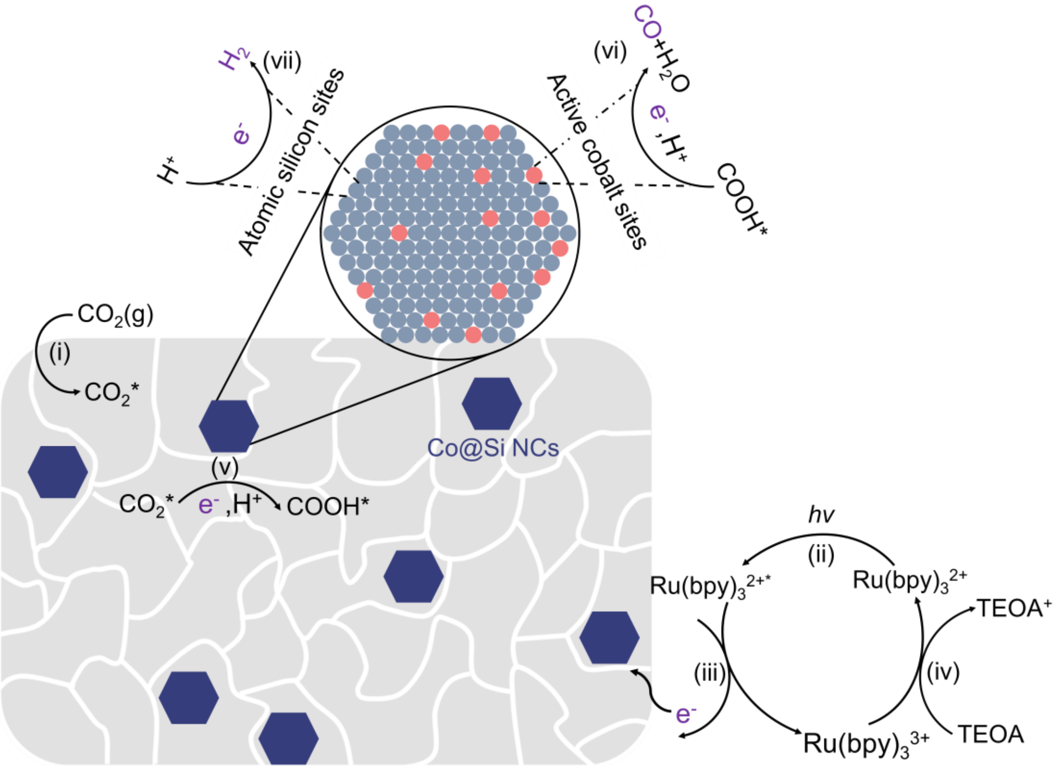

Supplementary Fig. 22 | Schematic illustration of the photocatalytic syngas production mechanism. The process was initiated by the physical adsorption of CO_2 gas on the catalyst (i). From steps (ii) to (iv) are the redox-active pathways using $Ru(bpy)_3^{2+}$ and TEOA as the photosensitizer and the sacrificial agent, respectively. The COOH* intermediate is expected to form from the interaction between proton and CO_2 molecules. The protons can be further reduced and form gaseous hydrogen (H_2) on the surface Si atoms, whereas the CO is generated from the active Co sites on the SiNC surfaces.

Supplementary Table 1 | Summary of ICP-OES results of Co@Si SACs prepared under different synthetic conditions.

Initial $CoCl_2 \cdot 6H_2O$ for sol-gel reaction (mmol)	Co concentration on Co@Si SAC (wt%)
0.2	0.4
0.4	0.5
2	1.4
4	3.4

Supplementary Table 2 | Summary of the elemental analysis results by EDX measurements on Co@Si SACs with various Co concentrations.

Sample	Atomic % (O)	Atomic % (Si)	Atomic % (Co)
0.4 wt%Co SAC	67.188	32.477	0.335
0.5 wt%Co SAC	55.701	43.834	0.465
1.4 wt%Co SAC	62.029	36.883	1.087
3.4 wt%Co SAC	45.471	52.544	1.985

Supplementary Table 3 | EXAFS fitting data at the Co K-edge of various Co@Si samples.

Co loading (wt%)	Scattering Path	Coordination Number	R (Å)	ΔE_0 (eV)	σ^2(Å^2)
0.4	Co-Si	2.5	2.24	-6.7	0.00347
0.5	Co-Si	3.8	2.26	-7.8	0.00357
1.4	Co-O	1.1	1.83	-9.0	0.00474
	Co-Si	4.0	2.31	-3.6	0.00496
3.4	Co-O	0.5	1.82	-9.0	0.00577
	Co-Si	3.1	2.31	-5.1	0.01991
	Co-Co	1.4	2.37	-0.1	0.01942

Supplementary Table 4 | Summary of the catalytic performance of the catalysts listed in Supplementary Fig. 18.

No.	Metal loading (wt%)	TON (reaction time)	Catalyst	Ref. in the Supplementary Information
1	0.2	678 (3 h)	Co/graphene	1
2	5	4.3 (2 h)	Co/g-CN_x	2
3	2.6	35 (2 h)	Co-ZIF-9/g-C_3N_4	3
4	0.8	155 (17 h)	Fe/C_3N_4	4
5	0.1	450 (30 h)	Fe/$CuInS_2$	5
6	0.1	280 (4 h)	Ni/ZnSe	6
7	2	6.5 (1 h)	Cu-CCN	7
8	2.4	200 (2 h)	Co/C_3N_4	8
9	0.1	421 (20 h)	Co/Bi_3O_4Br	9
10	8	1494 (1 h)	Fe−NO/NC	10
11	0.08	21.2 (4.5 h)	Ni/C_3N_4	11
12	5	11.5 (6 h)	Ni@MOF	12
13	0.01	1.45 (24 h)	Cu/TiO_2	13
**This work**	**1.4**	**19536 (6 h)**	**Co@Si SAC**	\

Note: CN_x = carbon nitride; CCN = crystalline carbon nitride; NC = nitrogen-rich carbon; MOF: metal-organic framework

Supplementary Table 5 | Summary of the catalytic performance of the catalysts listed in Fig. 4d.

Ref. in the manuscript	Metal loading (wt%)	EQE (%)	Catalyst type
50	0.2	0.4	Pd@P7
20	5	0.35	Al/Cu_2O
51	2.6	1.44	Cu_2O-Pt/SiC/IrO_x
52	0.8	0.36	Co/Bi_3O_4Br
15	0.1	0.4	Co/C_3N_4
53	0.1	0.5	Ru/ZnS
54	2	10	Cu/TiO_2
55	2.4	2	CuO/TiO_2
17	0.1	1.9	Ru/Ta_2O_5
56	8	1.5	Ru/C_3N_4
18	0.08	5.7	Ru/C_3N_4
57	5	5.2	Ru/C_3N_4
58	0.01	1.1	Ru(II) /C_3N_4
59	0.12	0.25	Co/C_3N_4
16	0.11	0.9	Co-ZIF-9/g-C_3N_4
60	4.4	0.8	Co/C_3N_4
61	14	4.2	Fe/C_3N_4
62	1.5	0.48	Ag/TaON
63	1.5	0.2	Ru/CN_x nanosheet
64	1.5	1	Ru/GaN:ZnO
**This work**	**3.4**	**10**	**Co@Si SAC**

Note: CN_x = carbon nitride; P7 = dibenzo[*b,d*]thiophene sulfone

Supplementary Note 1 | More details of the quantification of single Co atoms using electron microscopy techniques

Here, we discuss challenges in quantification of Co atoms using electron microscopy techniques in our study.

One can apply the quantitative analysis technique "atom counting" to quantitatively analyze both positions and numbers of atoms inside a nanomaterial[14]. Moreover, it is possible to capture an EELS signal of single atoms. However, it should be noted that colloidal single-atom catalysts are typically quite electron-beam sensitive and thus single-atoms can delocalize upon electron beam illumination. To reduce the electron beam damage, we have used graphene TEM grids (to reduce the background and potential electron beam damage) and relatively low beam current during our electron microscopy measurement. Still, possible structural changes induced by the electron beam may influence the reliability of the measurements when attempting to capture EELS signal or quantitative atom counting for single Co atom.

Another challenge that hampers the ability of locating single Co atoms is unavoidable electron-beam induced carbon contamination. As a mitigation, we applied activated charcoal based pre-clean treatment for the single-atom catalysts prior to TEM measurements to absorb isolated ligands and potential organic residuals[15]. Still, a rapid growth of carbon layer under electron beam leads to a continuously increasing background signal during EELS measurement. Moreover, the carbon contamination induced inhomogeneous background intensity, which makes it more challenging to achieve a reliable quantification of positions and numbers of single Co atom inside the catalysts.

Finally, the amount of Co atom is quite low, hence it is quite challenging to extract background (*e.g.*, carbon) to localize the EELS signal of those single atoms and therefore correlating with atomic positions based on EELS mapping.

We foresee that extra efforts on quantitative imaging analysis technique are needed to tackle challenges in quantification of atomic structure of beam-sensitive materials in the future.

Supplementary Note 2 | Functions of crystalline Si component on Co@Si SACs for CO_2RR.

To better elucidate the catalytic behaviour of the active Co sites, we removed the SiO_2 matrix and CoO_x to liberate Co@Si NCs by HF etching[16]. The colloidal Co@Si NCs were subsequently used for CO_2RR under the same reaction condition for 9 h (Supplementary Fig. 17). The yield of CO (200 μmol $g^{-1}\cdot h^{-1}$) is 18 times higher than the reaction with Co-free Si NCs but significantly lower than the Co@Si SAC sustained by porous SiO_2 matrix, indicating that the silicon-based Co@Si nanostructures play a crucial role in CO_2-to-CO conversion. More importantly, efficient CO_2RR performance can be achieved without applying additional photosensitizer. This may be attributed to the intense light absorption capability of c-Si, which enhances the photoelectron generation and the subsequent charge carrier separation on Co@Si SACs (Supplementary Fig. 16).

Supplementary Note 3 | Influences of the proton donor and the sacrificial reagent on the H_2/CO ratio in the syngas product.

We carried out control photocatalytic experiments with a varied amount of water and sacrificial reagent to investigate their impact on the product types and yields. Results show that the number of protons has a dominant influence on photocatalytic performance (Supplementary Fig. 19). The H_2/CO ratio can be tuned between 1 - 2 by varying the usage of H_2O (Supplementary Fig. 19a), indicating that water plays a key role generation of protons. We also found that the Co concentration of Co@Si SACs and the amount of sacrificial agent have a minor influence on the H_2/CO ratio (Fig. 4b and Supplementary Fig. 19b). We therefore conclude that the number of protons is more vital to the H_2/CO ratio in syngas production.

References

1. Gao C. et al. Heterogeneous single-atom catalyst for visible-light-driven high-turnover CO_2 reduction: The role of electron transfer. *Adv. Mater.* **30**, 1704624 (2018).

2. Lin, J, Pan, Z, and Wang, X. Photochemical reduction of CO_2 by graphitic carbon nitride polymers. *ACS Sustain. Chem. Eng.* **2**, 353 (2014).

3. Kas, R. et al. Electrochemical CO_2 reduction on Cu_2O-derived copper nanoparticles: controlling the catalytic selectivity of hydrocarbons. *Phys. Chem. Chem. Phys.* **16**, 14656 (2014).

4. Cometto, C. et al. A carbon nitride/Fe quaterpyridine catalytic system for photostimulated CO_2-to-CO conversion with visible light. *J. Am. Chem. Soc.* **140**, 7437 (2018).

5. Lian, S., Kodaimati, M. S., Weiss, E. A. Photocatalytically active superstructures of quantum dots and iron porphyrins for reduction of CO_2 to CO in water. *ACS Nano* **12**, 568 (2018).

6. Kuehnel, M. et al. ZnSe quantum dots modified with a Ni(cyclam) catalyst for efficient visible-light driven CO_2 reduction in water. *Chem. Sci.* **9**, 2501 (2018).

7. Li, Y. et al. Crystalline carbon nitride supported copper single atoms for photocatalytic CO_2 reduction with Nearly 100% CO Selectivity. *ACS Nano* **14**, 10552 (2020).

8. Huang, P. P. et al. Selective CO_2 reduction catalyzed by single cobalt sites on carbon nitride under visible-light irradiation. *J. Am. Chem. Soc.* **140**, 16042 (2018).

9. Di, J. et al. Isolated single atom cobalt in Bi_3O_4Br atomic layers to trigger efficient CO_2 photoreduction. *Nat. Commun.* **10**, 2840 (2019).

10. Li, Y. et al. Facile top-down strategy for direct metal atomization and coordination achieving a high turnover number in CO_2 photoreduction. *J. Am. Chem. Soc.* **142**, 19259 (2020).

11. Cheng, L. et al. Single Ni atoms anchored on porous few-layer g-C_3N_4 for photocatalytic CO_2 reduction: the role of edge confinement. *Small* **16**, 2002411 (2020).

12. Niu, K. et al. A spongy nickel-organic CO_2 reduction photocatalyst for nearly 100% selective CO production. *Sci. Adv.* **3**, e1700921 (2017).

13. Fang, B. et al. Hierarchical CuO–TiO_2 Hollow Microspheres for Highly Efficient Photodriven Reduction of CO_2 to CH_4. *ACS Sustain. Chem. Eng.* **3**, 2381 (2015).

14. Van Aert, S. et al. Three-dimensional atomic imaging of crystalline nanoparticles. *Nature* **470**, 374 (2011).

15. Li, C. et al. A simple method to clean ligand contamination on TEM grids. *Ultramicroscopy* **221**, 113195 (2021).

16. Clark, R. J. et al. From hydrogen silsesquioxane to functionalized silicon nanocrystals. *Chem. Mater.* **29**, 80 (2016).
